# Practical Notes on Surgical Diagnosis and Treatment

**Published:** 1911-01-28

**Authors:** 


### Practical Notes on Diagnosis and Treatment.

### Adduction and Abduction of the Foot.

With adduction is associated a high arch, with abduction a flattening of the arch. In adduction the toes of the boots curl up to form a crease across the "uppers"; these people are not always steady when standing, but it is the position of the quick and alert. In abduction the upper of the boot is long and has no crease across the toe, while the subjects stand firmly on their legs; it is the position of the slow, heavy, and inert. In the inactive abducted position the ankles tend to bend inward, forming the very common condition in children called weak ankles, the internal malleolus not being sufficient to prevent this. In these cases the muscles must be educated, and their action not replaced by special apparatus or boots.—*Mr. Edred Corner.*

### Hæmatoma of the Sterno-mastoid.

The usual cause of hæmorrhage in the sterno-mastoid is difficulty in labour. Three-fourths of the cases are undoubtedly associated with breech presentations, and the muscle which usually suffers is the right sterno-mastoid. In a moderate number of cases a hæmatoma was followed by the onset of torticollis. In a small proportion the hæmatoma disappeared and was not followed by the onset of torticollis. With regard to treatment, it is advisable to wait; for in many cases a mild degree of contraction will be overcome by manipulation and passive movements, and in other cases it seems that much of the contraction passes off spontaneously without any interference whatever. No question of surgical interference should be entertained in such a child under the second or third year of its age.—*Mr. A. H. Tubby.*

### Enema of Asafœtida.

A useful remedy for abdominal distention is the old-fashioned asafœtida enema. It is not so irritating as turpentine, and often brings away much flatus, and so relieves for a short time, at any rate, the cardiac and respiratory embarrassment, which is so urgent when the upper part of the abdomen is implicated.

### The Nauheim Treatment.

The generally accepted theory of the action of the Nauheim baths is as follows: The stimulation of the nerve-endings by the action of the $CO_2$ given off causes dilatation of the peripheral circulation, so causing a larger arterial "bed," and at the same time reducing the amount of work of the central cardiac mechanism. This peripheral dilatation is produced without raising the temperature of the baths, thus avoiding any disturbance of thermogenesis, which is both injurious and exhausting, as it increases tissue waste.

### Icterus Neonatorum.

This is not at all an uncommon condition: something like 60 to 80 per cent. of children after birth present signs of jaundice. In a very large proportion the jaundice passes away without leaving any traceable ill-effects; but in some cases the jaundice deepens, and then it becomes a sign of serious import. It is a striking point with regard to the jaundice of newly-born children that the conjunctivæ and urine are not coloured, unless the jaundice is of a very intense description, whereas in icteric adults there is always a yellowness of the conjunctivæ and an alteration of the colour of the urine.—*Mr. A. H. Tubby.*

